# Absolute mortality risk assessment of COVID-19 patients: the Khorshid COVID Cohort (KCC) study

**DOI:** 10.1186/s12874-021-01340-8

**Published:** 2021-07-14

**Authors:** Hamid Reza Marateb, Maja von Cube, Ramin Sami, Shaghayegh Haghjooy Javanmard, Marjan Mansourian, Babak Amra, Forogh Soltaninejad, Mojgan Mortazavi, Peyman Adibi, Nilufar Khademi, Nastaran Sadat Hosseini, Arash Toghyani, Razieh Hassannejad, Miquel Angel Mañanas, Harald Binder, Martin Wolkewitz

**Affiliations:** 1 Biomedical Engineering Department, Engineering Faculty, University of Isfahan, Isfahan, Iran; 2 Biomedical Engineering Research Centre (CREB), Automatic Control Department (ESAII), Universitat Politècnica de Catalunya-Barcelona Tech (UPC)Building H, Floor 4, Av. Diagonal 647, 08028 Barcelona, Spain; 3 Institute of Medical Biometry and Statistics, Faculty of Medicine and Medical Center, University of Freiburg, Freiburg, Germany; 4 Department of Internal Medicine, School of Medicine, Isfahan University of Medical Sciences, Isfahan, Iran; 5 Applied Physiology Research Center, Cardiovascular Research Institute, Isfahan University of Medical Sciences, Isfahan, Iran; 6 Department of Epidemiology and Biostatistics, School of Health, Isfahan University of Medical Sciences, Isfahan, Iran; 7 Bamdad Respiratory Research Center, Isfahan University of Medical Sciences, Isfahan, Iran; 8 The Respiratory Research Center, Pulmonary Division, Department of Internal Medicine, School of Medicine, Isfahan University of Medical Sciences, Isfahan, Iran; 9 Isfahan Kidney Diseases Research Center, Isfahan University of Medical Sciences, Isfahan, Iran; 10 Isfahan Gastroenterology and Hepatology Research Center (lGHRC), Isfahan University of Medical Sciences, Isfahan, Iran; 11 School of Medicine, Isfahan University of Medical Sciences, Isfahan, Iran; 12 Isfahan Cardiovascular Research Center, Cardiovascular Research Institute, Isfahan University of Medical Sciences, Isfahan, Iran; 13 Biomedical Research Networking Center in Bioengineering, Biomaterials, and Nanomedicine (CIBER-BBN), Madrid, Spain

**Keywords:** Cause-specific hazard regression, COVID-19, Mortality, Prognosis, Risk assessment, Risk chart

## Abstract

**Background:**

Already at hospital admission, clinicians require simple tools to identify hospitalized COVID-19 patients at high risk of mortality. Such tools can significantly improve resource allocation and patient management within hospitals. From the statistical point of view, extended time-to-event models are required to account for competing risks (discharge from hospital) and censoring so that active cases can also contribute to the analysis.

**Methods:**

We used the hospital-based open Khorshid COVID Cohort (KCC) study with 630 COVID-19 patients from Isfahan, Iran. Competing risk methods are used to develop a death risk chart based on the following variables, which can simply be measured at hospital admission: sex, age, hypertension, oxygen saturation, and Charlson Comorbidity Index. The area under the receiver operator curve was used to assess accuracy concerning discrimination between patients discharged alive and dead.

**Results:**

Cause-specific hazard regression models show that these baseline variables are associated with both death, and discharge hazards. The risk chart reflects the combined results of the two cause-specific hazard regression models. The proposed risk assessment method had a very good accuracy (AUC = 0.872 [CI 95%: 0.835–0.910]).

**Conclusions:**

This study aims to improve and validate a personalized mortality risk calculator based on hospitalized COVID-19 patients. The risk assessment of patient mortality provides physicians with additional guidance for making tough decisions.

## Background

The ongoing coronavirus disease 2019 (COVID-2019) pandemic changed priorities all over the world. More than 119 million confirmed infections and over 2.6 million deaths worldwide have been reported [1, 2]. Approximately 20% of such confirmed cases were severe, among which most were admitted in the intensive care unit (ICU) and required early intubation and mechanical ventilation. The health systems thus face financial and facility challenges in light of managing this condition. As the pandemic continuous, hospitals seek effective methods for managing such severe patients. Present guidelines on COVID-19 treatment identify ICU admission, ventilation, and mortality risk as typical outcomes in high-risk patients and consider these patients as potential candidates for medical treatment [3, 4]. Many studies reported the clinical characteristics and outcomes of COVID-19 patients, but few research studies focused on the risk assessment of outcomes [5, 6]. Personal risk profiles can help physicians make the correct decision for optimal patient treatment and hospital capacity management.

The risk assessment procedure and the resulting risk charts have been well documented in community-based cardiovascular Cohorts [7–11]. In such studies, the risk of having an event by the end of the cohort follow-up is estimated using the time-to-event analysis based on Cox proportional hazard regression [12]. By contrast, the analysis of in-hospital data requires special attention. Standard Cox regression models lead to inaccurate results when competing events exist [13]. For example, discharge alive is a competing event when hospital death is the event of interest. Such information should not be ignored since it results in an incomplete reflection of treatment effects [14] and creates competing risk bias. Moreover, standard logistic regression could not be used when some active cases are still hospitalized at the last follow-up date [13, 15]. In fact, excluding such patients creates selection bias.

Recently, machine learning methods were used for survival analysis. Methods, such as Random Survival Forests (RSF), were compared with standard Cox regression models [16]. Although RSF could select significant nonlinear interactions to improve the discrimination ability, they do not provide direct clinical interpretation information and should be further studied before the clinical application [17, 18]. Also, time-to-event data analysis could not be performed appropriately using classification systems, in which the event-of-interest is only used, and time-to-event data is discarded with the same reason mentioned above for the standard logistic regression.

This paper introduces an absolute cause-specific risk regression approach to perform risk assessment [19–21] for patients hospitalized with COVID-19. The event-of-interest is death in the hospital, and discharge alive is considered as the competing event. To demonstrate the methodology, the Khorshid COVID-19 Cohort (KCC) dataset [22] with 630 patients is used. To the best of our knowledge, this is the first study in which absolute risk assessment is performed on in-hospital COVID-19 patients considering the competing events. We also provide a death risk chart as a simple tool for clinical applications.

## Methods

### Absolute risk estimation for hospital mortality

First, we introduce the absolute risk estimation approach, which is used for the risk assessment.

We denote D the event outcome, where D = 1 if the event of interest (e.g., hospital death) occurred, and D = 2 if the competing event (e.g., discharge alive) happened. Then, the probability (i.e., absolute risk) that an event of type 1 (i.e., D = 1) occurred by time *t* is given by the Eq. (1) [20, 21]:

1 $${F}_{1}\left(t|x,z\right)={\int }_{0}^{t}S\left(s-|x,z\right){\lambda }_{1,z}\left(s|x\right)ds$$

where $s-$ denotes the right-sided limit, $S\left(s-|x,z\right)$ is the conditional event-free survival function, ${\lambda }_{1,z}\left(s|x\right)$ is the hazard of the event of interest dependent on the baseline covariate vector **x**, and **z** is a set of strata variables. The event-free survival function,$S(s|x,z)$, is the probability to be still hospitalized at time s and thus depends on the patients’ length of hospital stay which is directly linked to both the death hazard, ${\lambda }_{1,z}\left(s|x\right),$ and the discharge hazard, ${\lambda }_{2,z}\left(s|x\right).$ This is in contrast to the classical time-to event settings with only one event of interest (here: death) and without competing events (here: discharge). Thus, the absolute risk of hospital mortality is preliminary determined by the death hazard but also by the discharge hazard. As many clinical factors and biomarkers are associated with the discharge hazard, it is therefore necessary to use competing risk methodology to identify potential predictors for hospital mortality.

To estimate ${F}_{1}\left(t|x,z\right)$, we first need to estimate the event-free survival function. It is done by estimating both cause-specific hazards and then using the product integral estimator [21]. The stratified Cox regression model for cause *j, j* = *1 or 2* [21, 23], is given by the Eq. (2):

2 $${\lambda }_{0j,z}\left(t|x\right)={\lambda }_{0j,z}(t){e}^{x{\beta }_{j}}$$

where β_j_ is the vector of log-hazard ratios of the covariate vector x and λ_0j,z_(t) is the baseline hazard function.

Thus, the absolute risk estimation includes estimating the cause-specific hazards for both event types and then calculating the integral Eq. (1).

### Obtaining the risk chart

**Fig. 1:**
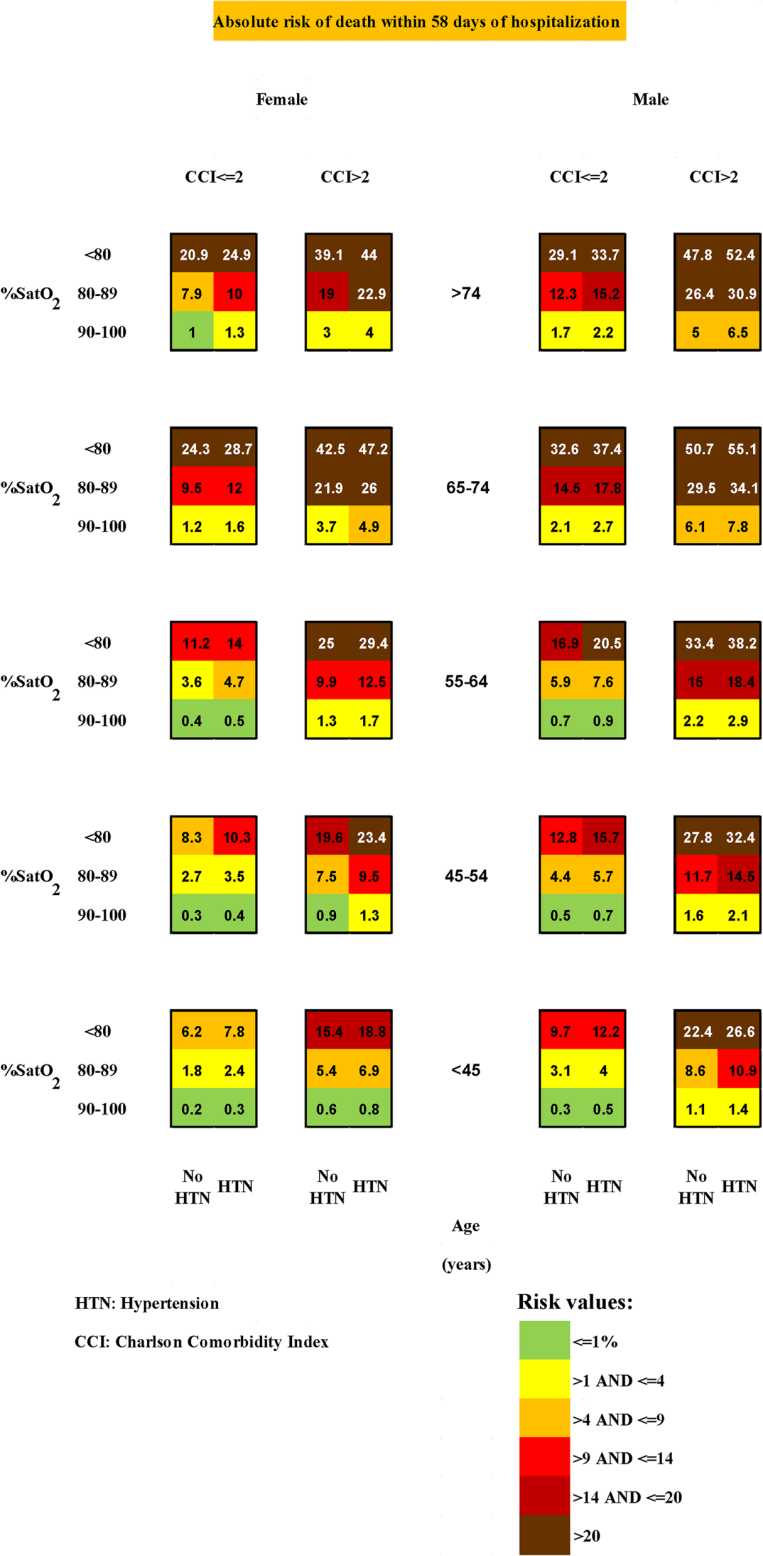
The absolute mortality risk chart for COVID-19 hospitalized patients based on sex, age, oxygen saturation, hypertension, and Charlson Comorbidity Index

We use the absolute risk estimation procedure to obtain a detailed risk chart for patients hospitalized for COVID-19 (see Fig. 1). First, two cause-specific Cox proportional hazards models for the outcome events death in the hospital and discharged alive are fitted. It is done by using the “*csc”* function of the “*riskRegression”* package in R [21, 24]. The cause-specific hazard ratios show how the risk factors affect each hazard rate. Both cause-specific hazard ratios have to be considered to conclude the effects of the risk factors on the absolute risk. Thus, even if a variable does not affect the death hazard, it may indirectly affect hospital mortality by in- or decreasing the discharge hazard. By contrast, the subdistribution hazard ratio combines the direct and the indirect effects in a single coefficient. To show how variables may indirectly influence hospital mortality, we additionally estimate the subdistribution hazard for the outcome of death. It is done using the “*crr”* function of the “*cmprsk”* package in R [25–27].

Nonetheless, the absolute risk approach is based on the two cause-specific hazards, as was explained above. Thus, we apply the “*predict”* function on the *csc*-object to obtain the estimates of ${F}_{1}\left(t|x,z\right)$ for all patient groups. For each patient, we obtain an individual risk based on the baseline covariates of the patients. Finally, the risk chart as shown in Fig. 1 can be constructed. By color-coding, high-risk and low-risk patients are directly identifiable from the chart.

The area under the curve (AUC) of the receiver operator curve (ROC), defined in Eq. (3) [28–30], could be used to assess accuracy for discrimination between patients discharged alive and dead:

3 $$AUC=\frac{1}{m\times n}\sum_{i=1}^{m}\sum_{j=1}^{n}\varphi \left({P}_{i},{P}_{j}\right)$$

where *m* is the number of patients who died, *n* is the number of patients discharged alive, *P*_*i*_ and *P*_*j*_ are the estimated mortality risks of the data points *i* and *j*. The kernel $\varphi$ is defined in Eq. (4).

4 $$\varphi \left({P}_{i},{P}_{j}\right)=\left\{\begin{array}{cc}1& {P}_{i}>{P}_{j}\\ 0.5& {P}_{i}={P}_{j}\\ 0& {P}_{i}<{P}_{j}\end{array}\right.$$

It is done using the “colAUC” function of the “caTools” package in R [31, 32].

Results were reported as mean ± standard deviation (for interval variables) and frequencies (for categorical variables). All data processing was performed using R version 4.0.3 [33].

### Dataset and variable selection

In our study, the Khorshid COVID-19 Cohort (KCC) dataset [22, 34] was used. It is a hospital-based open cohort from Isfahan, which was a hot outbreak zone in central Iran. COVID-19 patients were admitted to the Khorshid referral hospital in Isfahan from February 2020 until September 2020. The patient recruitment phase finished at the end of August 2020, and the follow-up continues until the end of August 2021. The patients are followed up for the first, fourth, 12^th^ weeks, and the first year after discharge. In total, 630 COVID-19 patients were enrolled in our study.

Among the recorded data for each COVID-19 patient, the baseline parameters sex, age, hypertension, oxygen saturation (SaO2) at hospital admission, and Charlson Comorbidity Index (CCI) [35, 36] were used as the non-lab input measurements to the model. Such parameters were selected based on clinical knowledge and previous papers published in the literature on the COVID-19 clinical outcome prediction [37, 38]. Age was categorized as less than 45 (years) (B0), 45–54 (B1), 55–64 (B2), 65–74 (B3), and more than 75 (B4) as in Petrilli et al. [39]. Oxygen saturation was categorized as more than or equal to 90% (A0), 80–89 (A1), and less than 80 (A2) by merging some SaO2 sub-categories as mentioned by Mejía et al. [38]. CCI was dichotomized as low (CCI < 3) or high (CCI ≥ 3). Such an optimal cut-off was estimated to minimize the (Error-Rate) ER-criteria of the Receiver operating characteristic curve (ROC) [40] for optimal mortality discrimination (AUC = 0.767 [CI 95%: 0.697–0.837]). Such a threshold previously was used in the literature for risk stratifications of hospitalized COVID-19 patients [41]. A reference group is considered with the following categories: sex (Female), age (B0), oxygen saturation (A0), and low CCI. Time-to-event was considered until hospital discharge, and the status at discharge (dead or alive) is taken into account. The risk assessment procedure was performed for the time from admission until 58 days of hospitalization.

## Results

Among 630 patients, 38.7% were female. The average age of the admitted patients was 57.1 ± 15.4 years. The average of the CCI was 2.3 ± 2.1. 34.9% of the participants had hypertension. Forty-five patients died in the hospital. The descriptive statistics of the admitted patients categorized by the vital state at discharge are listed in Table 1. The cause-specific hazard ratios and their 95%-CIs are shown in Table 2. The subdistribution hazard ratios and their CI 95% for mortality based on the Fine and Gray model [26], with the competing event of discharge alive, are listed in Table 3. For example, we find that low oxygen saturation significantly increases the death hazard rate and decreases the discharge hazard. If it drops below 80%, the death hazard is more than five times higher than for patients in the reference group. At the same time, the discharge hazard is 40% lower. The subdistribution hazard ratio quantifies the combined effect: patients with 80% oxygen saturation or less have a more than ten times higher risk of death in the hospital. This effect is not only explained by the increased death hazard but also by the decreased discharge hazard. Due to the decreased discharge hazards, the patients stay longer in the hospital and therefore longer at risk and thus also at higher risk of dying in the hospital within 58 days.

**Table 1:** The baseline characteristics of 630 patients, discharged alive or dead

Parameter	Discharged alive *n* = 585	Dead *n* = 45
Sex (male)	359 (61.4)	27 (60.0)
Age	56.1 ± 15.1	70.0 ± 13.4
Oxygen saturation	90.0 ± 6.1	80.1 ± 12.6
CCI	2.1 ± 2.0	4.2 ± 2.2
Hypertension	193 (33.0)	27 (60.0)

*CCI* Charlson Comorbidity Index. Data are Mean ± SD or numbers (%)

**Table 2:** The multivariable Cox proportional hazard regression model for mortality and hospital discharge

Parameter	Category	Death Hazard Ratio	Discharge Hazard Ratio
Sex			
	Female, ref	-	-
	Male	1.52 [0.77–2.99]	0.93 [0.78–1.11]
Age (years)			
	< 45, ref	-	-
	45–54	1.02 [0.14–7.33]	0.82 [0.64–1.05]
	55–64	1.81 [0.36–9.07]	0.93 [0.73–1.19]
	65–74	3.84 [0.78–18.87]	0.77 [0.57–1.04]
	> 74	2.74 [0.51–14.80]	0.73 [0.50–1.05]
Oxygen Saturation (%)			
	90–100, ref	-	-
	80–89	3.19 [1.33–7.65]	0.57 [0.47–0.69]
	< 80	5.48 [2.18–13.78]	0.38 [0.27–0.53]
CCI			
	0–2, ref	-	-
	≥ 3	2.10 [0.90–4.86]	0.80 [0.61–1.05]
Hypertension		1.14 [0.55–2.37]	0.92 [0.75–1.12]

*CCI* Charlson Comorbidity Index

**Table 3:** The multivariable subdistribution hazards model for hospital mortality

Parameter	Category	Subdistribution Hazard Ratio	CI 95%	*p*-value
Sex				
	Female, ref	-	-	-
	Male	1.41	0.72–2.78	0.320
Age (years)				
	< 45, ref	-	-	-
	45–54	0.91	0.14–6.10	0.920
	55–64	1.53	0.32–7.39	0.600
	65–74	3.49	0.70–17.56	0.130
	> 74	2.40	0.42–13.72	0.330
Oxygen Saturation (%)				
	90–100, ref	-	-	-
	80–89	5.37	2.27–12.74	< 0.001
	< 80	10.94	3.90–30.66	< 0.001
CCI				
	0–2, ref	-	-	-
	≥ 3	2.18	0.94–5.05	0.068
Hypertension		1.41	0.72–2.76	0.320

*CCI* Charlson Comorbidity Index

**Fig. 2:**
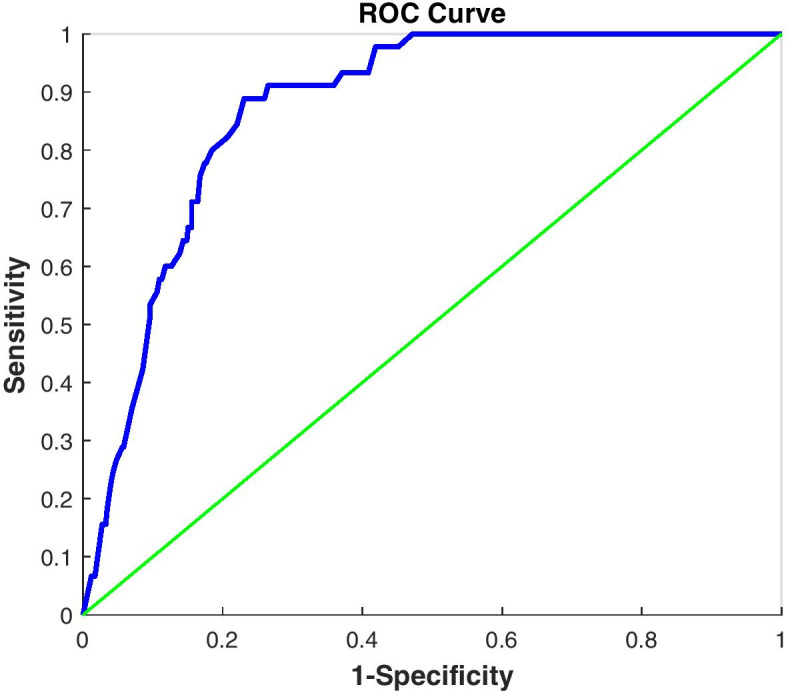
The Receiver operating characteristic (ROC) curve of the proposed risk assessment method for discrimination between the dead and discharged alive patients. The parameters Sensitivity (Specificity) are the proportion of patients who died (discharged alive) that were correctly identified by the model

The absolute mortality risk was then calculated within 58 days of hospitalization of COVID-19 patients. Its color-coded risk chart is provided in Fig. 1. The proposed risk assessment method had an excellent accuracy (AUC = 0.872 [CI 95%: 0.835–0.910]). The ROC of the proposed system is shown in Fig. 2.

The risk chart reflects the combined results of the two cause-specific hazard ratios. We find that the risk of hospital death within each group is about ten times higher for patients with an oxygen saturation lower of 80%.

## Discussion

Suitable identification of hospitalized COVID-19 patients at high risk of mortality can significantly improve resource allocation and patient management within hospitals. This study aims to improve and validate a personalized mortality risk calculator based on hospitalized COVID-19 patients. The risk assessment of patient mortality provides physicians with additional guidance for making tough decisions. The presented tool showed a very good accuracy. In this model, baseline clinical parameters collected at hospital admission were used. It requires commonly available demographic and comorbidity data. Also, the oxygen saturation level could be measured using a traditional pulse oximeter. Thus, it does not require advanced blood testing and could be considered as a non-laboratory-based risk chart. The proposed risk assessment procedure is not limited to the input factors used in this paper. In fact, it could be applied to any input variables such as repeated COVID-19 infection, vaccination category, and body mass index.

Predicted mortality increased for patients with low oxygen saturation, corroborating findings that link hypoxemia to mortality [42], as well as the observed prevalence of shortness of breath in severe patients [43]. This measurement additionally serves as a signal of respiratory distress, and respiratory failure has been found clinically as one of the significant causes of COVID-19 mortality [44]. It can also appear in silent hypoxia cases where shortness of breath is not observed [45]. Age was another critical determinant of mortality in the model: older patients have higher mortality risk, as observed in the retrospective patient analysis [46] and subsequently reflected in public health guidance [47]. Similar to our study, it was shown in the literature that a higher CCI score is significantly associated with mortality and disease severity in COVID-19 patients [41, 48].

Moreover, the increase in mortality risk for patients with elevated blood pressure levels is consistent with the reports in other studies [49, 50]. Such findings could have been expected a priori as logical, but the proposed assessment permits quantifying them as a function of time after patient admission. In our analysis, a dichotomized version of CCI was used (CCI < 3 vs. CCI ≥ 3). However, more categories of CCI were used in the literature, such as: CCI = 0, CCI score of 1–2, and CCI score of ≥ 3 [41]. Due to the collinearity between age and CCI, our model consisted of a dichotomized version of CCI.

In our dataset, no active patients were in the hospital, and follow-up was complete for hospital mortality. Thus, in principle, the logistic regressions could be alternatively used instead of the absolute risk regression model in our data situation. However, it does not account for time-to-event and censoring. It could only display effects on a cumulative incidence function on a plateau [15]. Moreover, since we aim to use the proposed risk assessment procedure in dynamic situations and integrate it into the hospital information system (HIS), only predictions based on cause-specific regression could be used as there are always active cases in the hospital proceeding time frames.

The example of oxygen saturation categories demonstrated the possible incomplete picture of risk assessment if the competing risk of discharge alive is ignored. In the Cox regression, where discharge alive is not considered (cause-specific hazard ratio of death Tables 2 and 3), the effect of oxygen saturation would have been estimated to be 5.48. However, considering the competing risk, discharge alive showed an indirect effect on hospital mortality via an increased length of stay. Thus, in contrast to a classical survival situation without competing risks, the cause-specific hazard ratio has no one-to-one relationship with the absolute risk. Estimating the subdistribution hazard ratio combined direct and indirect effects and showed that the mortality risk is increased tenfold rather than only fivefold. Such differences affect the estimated mortality risks.

The available data limit our prediction model. Considering more comprehensive variables such as IL-6 levels, D-Dimer, and radiographic diagnosis, may yield more accurate results in the laboratory-based risk charts. While the proposed model focused on the COVID-19 dataset at the national level, some studies considered multi-center region-specific data to measure how major risk factors identify the mortality rate [51]. Furthermore, the variability in treatment protocols across countries and individual organizations might lead to different results [22, 52]. External validation of the proposed risk assessment method is the focus of our future work.

## Conclusions

In conclusion, we provided a personalized mortality risk chart based on hospitalized COVID-19 patients to provide physicians with additional guidance for making tough decisions.

## Data Availability

The datasets used and/or analyzed during the current study are available from the corresponding author on reasonable request.

## Abbreviations

AUC: Area under the curve
CCI: Charlson Comorbidity Index
CI: Confidence Interval
COVID-2019: Coronavirus disease 2019
HR: Hazard Ratio
HIS: Hospital information system
ICU: Intensive care unit
KCC: Khorshid COVID Cohort
SaO2: Oxygen saturation
RSF: Random Survival Forests
ROC: Receiver operator curve
